# Alkamide Content and Localization in *Heliopsis longipes* Cypselae, Obtained via Fluorescence and Double-Multiphoton Microscopy

**DOI:** 10.3390/molecules29235651

**Published:** 2024-11-29

**Authors:** Juan Vázquez-Martínez, Jorge Molina-Torres

**Affiliations:** 1 Departamento de Ingeniería Química, TecNM/ITS Irapuato, Silao-Irapuato Km 12.5 El Copal, Irapuato CP 36821, GTO, Mexico; juan.vm@irapuato.tecnm.mx; 2 Departamento de Biotecnología y Bioquímica, Cinvestav Irapuato, Km. 9.6 Libramiento Norte Carretera Irapuato-León, Irapuato CP 36821, GTO, Mexico

**Keywords:** affinin, alkamides, GC-EIMS, *Heliopsis longipes* seeds, multiphoton microscopy, spilanthol

## Abstract

The alkamide content and specific tissue localization in the cypselae of *Heliopsis longipes* were investigated using gas chromatography–electron ionization mass spectrometry (GC-EIMS) and multiphoton fluorescence microscopy (MPFM). GC-EIMS analysis identified two olefinic alkamides: affinin (spilanthol) and *N*-2-methylbutyl-2*E*,6*Z*,8*E*-decatrienamide. Microscopic analysis revealed that alkamides are localized within the cotyledons, and specifically compartmentalized in lipid bodies, highlighting their spatial organization. The linear unmixing of fluorescence emission fingerprints showed that affinin exhibits autofluorescence at 693 nm, corresponding to the red spectral region. This emission is attributed to the conjugated double bonds in its acyl chain. This study is the first to report on the presence and precise localization of alkamides in the cypselae of *H. longipes.*

## 1. Introduction

Alkamides are a class of low-molecular-weight (<400 Da) plant-derived natural compounds characterized as *N*-substituted α-unsaturated acyl amides. These compounds typically consist of an α-unsaturated C8–C18 acyl chain attached to an amine through the amide bond. The most common acyl chain is a C10 structure, which may feature one double bond (*E*) or two conjugated (*E*,*E* or *E*,*Z*) double bonds. These double bonds can be positioned at the α-, intermediate, or methyl-ω sites, generally starting at an even-numbered carbon position [1]. *H. longipes* is a plant species known for producing alkamides predominantly in its roots. The primary alkamide found in this species is the *N*-isobutyl-2*E*,6*Z*,8*E*-decatrienamide, commonly referred to as affinin or spilanthol. Structurally, this compound features a C10 acyl chain with conjugated double bonds at the 2*E*, 6*Z*, and 8*E* positions, which is linked to an amine via an amide bond. Other (less abundant) *H. longipes* root alkamides include *N*-2-methylbutyl-2*E*,6*Z*,8*E*-decatrienamide and the bornyl ester of C10-2*E*,6*Z*,8*E* carboxylic acid [2]. It is well-established that, in addition to the roots, other plant structures, particularly seeds, also accumulate alkamides. However, the specific alkamides present in seeds and their distribution patterns remain uncharacterized.

Alkamides represent a structurally diverse group of natural compounds characterized by their varying configurations, including aromatic, polysubstituted, polyunsaturated, and dimeric structures [1]. These compounds are typically biosynthesized through enzymatic reactions involving the conjugation of acyl chains with amino acid-derived amino-moieties [3,4]. The resulting structural diversity contributes to their wide range of biological activities. Studies have demonstrated that these compounds exhibit significant bioactivity across a broad spectrum of plant and animal pathogens, including viruses, bacteria, and fungi [5]. Additionally, they possess pharmacological properties that affect animal systems, including humans, with reported activities including anti-inflammatory, analgesic, and immunomodulatory effects [6]. Thus, it is important to further the study of the distribution and synthesis of alkamides in plants.

Multiphoton fluorescence microscopy (MPFM), based on the simultaneous absorption of two or more photons by a fluorophore, has become a valuable technique in modern research due to its capacity for the real-time visualization of individual cells and their metabolites within intact tissues. This approach utilizes nonlinear optical contrast, enabling deep tissue penetration with minimal phototoxicity. As a non-invasive imaging modality, MPFM is recognized as a promising tool for “optical biopsy”, facilitating the examination of cellular functions in living systems [7,8,9].

The autofluorescence of plant secretory cells offers a powerful means of characterizing and localizing compounds with pharmaceutical relevance, particularly in the study of medicinal herbs. It is well established that secondary metabolites in plants, including those with pharmacological properties, can fluoresce, under UV light, in the visible spectral region [7,10,11]. This method, which requires no prior sample preparation, effectively distinguishes multiple endogenous fluorophores in living tissues [7,10,11]. Leveraging these fluorescence properties, we developed an imaging approach using MPFM to assess the localization of alkamides in seeds from *H. longipes*. To further validate the MPFM results, a gas chromatography–electron impact mass spectrometry (GC-EIMS) analysis was conducted. Thus, this study aims to elucidate the distribution of alkamides in *H. longipes* cypselae cells through fluorescence imaging using a two-photon microscope to obtain a better understanding of the accumulation patterns and potential physiological roles of these compounds.

## 2. Results and Discussion

### 2.1. Cypselae Dissection

**Figure 1:**
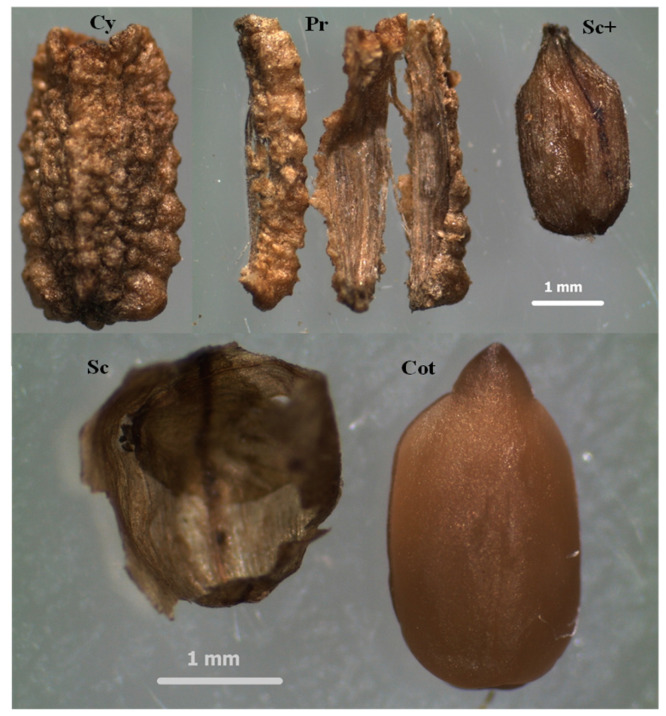
Structures of *H. longipes* cypselae (Cy) used for alkamides analyses: pericarp (Pr), seedcoat more cotyledon (Sc+), seedcoat (Sc), and cotyledon (Cot).

The term “cypsela” refers to a specific type of fruit, including that of *H. longipes*, commonly referred to as “seeds”. Unlike an achene, a cypsela is characterized by an additional layer, the perianth, covering the pericarp, which results from the inferior position of the ovary [12]. A cypsela consists of the pericarp (the external protective layer of the seed), the seed coat, and the seed (cotyledons) itself [13]. Using a stereo-dissecting microscope, the cypselae (Cy) were dissected into the pericarp (Pr), seed coat (Sc), and cotyledon (Cot), which contains the embryo (Figure 1). Cypselae contain a seed coat, pericarp, and cotyledon, comprising 9.4%, 37%, and 53.5%, respectively.

### 2.2. GC-EIMS Quantification of Cypselae Alkamide Content

**Figure 2:**
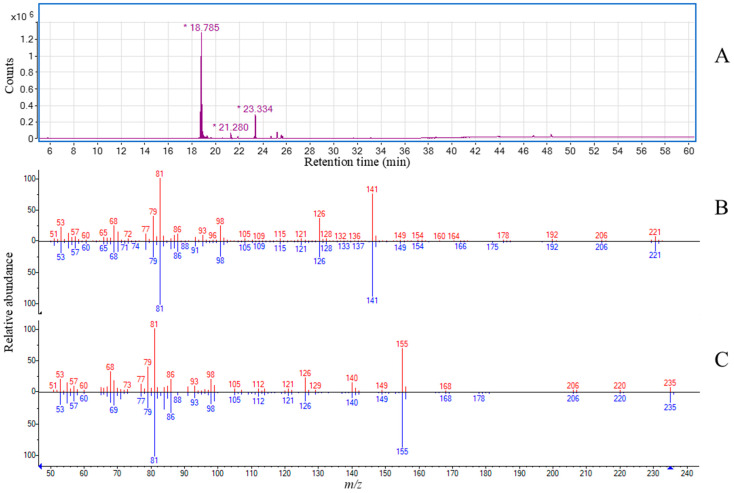
GC-EIMS analysis of *H. longipes* Cot ethanolic extract. (**A**) Total ion chromatogram of Cot extract; the components retained at *18.785, *21.280, *23.334 min are affinin, *N*-2-methylbutyl-2*E*,6*Z*,8*E*-decatrienamide, and farnesol acetate, respectively. (**B**) *N*-isobutyl-*2E,6Z,8E*-decatrienamide (affinin) mass spectrum; comparison between the component retained at 18.785 (red) and the authentic compound (blue). (**C**) *N*-2-methylbutyl-2*E*,6*Z*,8*E*-decatrienamide mass spectrum; comparison between the component retained at 21.280 (red) and the authentic compound (blue).

The GC-EIMS chromatogram of ethanolic extract from each dissected Cypsela (Cy) tissue, containing alkamides, is presented in Figure 2. For the identification and monitoring of alkamides, the fragment ion at *m*/*z* 81, corresponding to the position of the conjugated double bonds (C_6_H_9_), was used. Two alkamides were identified in the cotyledon (Cot) extract, with retention times (Rt) of 18.785 and 21.280 min. The component at Rt = 18.785 min exhibited a molecular ion [M+H]^+^ at 221 *m*/*z*, consistent with the molecular formula C_14_H_23_NO. The fragmentation pattern was as follows: *m*/*z* (relative intensity): 81 (999), 141 (950), 41 (345), 79 (240), 126 (239), 98 (234), 53 (178), 68 (164), 69 (159), and 86 (156). A comparison of this mass spectrum with the NIST database showed a 95% similarity with *N*-isobutyl-2*E*,6*Z*,8*E*-decatrienamide, also known as affinin or spilanthol, which is the major alkamide that was previously isolated and characterized from *H. longipes* roots [14]. The alkamide detected at Rt = 21.280 min was identified as *N*-2-methylbutyl-2*E*,6*Z*,8*E*-decatrienamide, with a molecular formula of C_15_H_25_NO. Its molecular ion [M+H]^+^ was 235 *m*/*z*, and the fragmentation pattern was as follows: *m*/*z* (relative intensity): 81 (999), 155 (850), 41 (367), 86 (299), 43 (257), 84 (248), 79 (229), 69 (196), 53 (186), and 98 (175). Both alkamides were found exclusively in the cotyledon (Cot) tissue and were absent in other structures, such as the pericarp (Pr) and seed coat (Sc), indicating a tissue-specific distribution of alkamides in *H. longipes* cypselae. Quantification revealed that affinin was the predominant alkamide in the cotyledon, present in 140 mg/g of fresh tissue, while *N*-2-methylbutyl-2*E*,6*Z*,8*E*-decatrienamide was present at a lower concentration of 0.5 mg/g of fresh tissue. In addition, the ethanolic extract of Cot has other non-alkamide compounds such as the farnesol acetate, retained at 23.334 min.

### 2.3. Microscopic Analysis of H. longipes Cypsela

**Figure 3:**
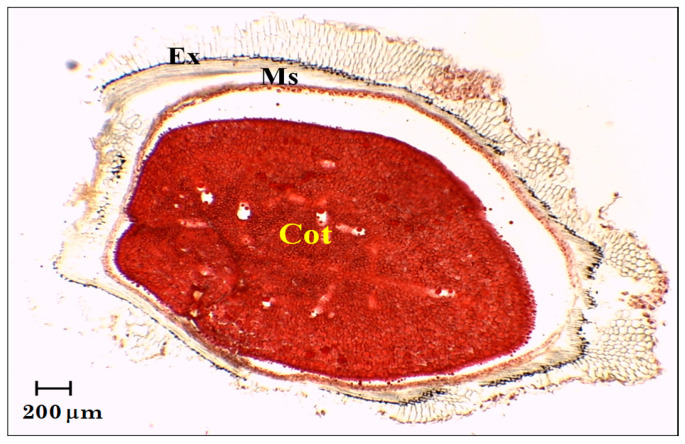
Transversal slice of *H. longipes* cypsela stained with NR (NR) observed using an optical microscope (10×). Cot = cotyledon; Ms = seedcoat; Ex = pericarp.

Affinin and related alkamides are recognized as olefinic compounds, characterized by their solubility in non-polar solvents. Consequently, it is likely that alkamides accumulate within the lipid bodies of plant tissues. In members of the Heliantheae tribe, cotyledons often contain lipid droplets that serve as energy reserves. These lipid bodies are commonly found in various plant cells and are not limited to storage tissues such as seeds, as is frequently assumed [15]. To investigate the presence of lipid bodies in the cotyledons of *H. longipes*, cypselae were sectioned using a microtome, and the slices were stained with NR dye. Lipid droplets within the cotyledons, which were stained red, are depicted in Figure 3. These findings can be correlated with the data obtained from GC-EIMS, suggesting that the specific localization of alkamides in the cotyledons corresponds to their accumulation in lipid droplets. The lipid bodies exhibited a typical spherical morphology, consistent with observations in other seeds, such as almond [16].

### 2.4. Multi-Photon Microscopy Localization of Affinin in H. longipes Cotyledons

**Figure 4:**
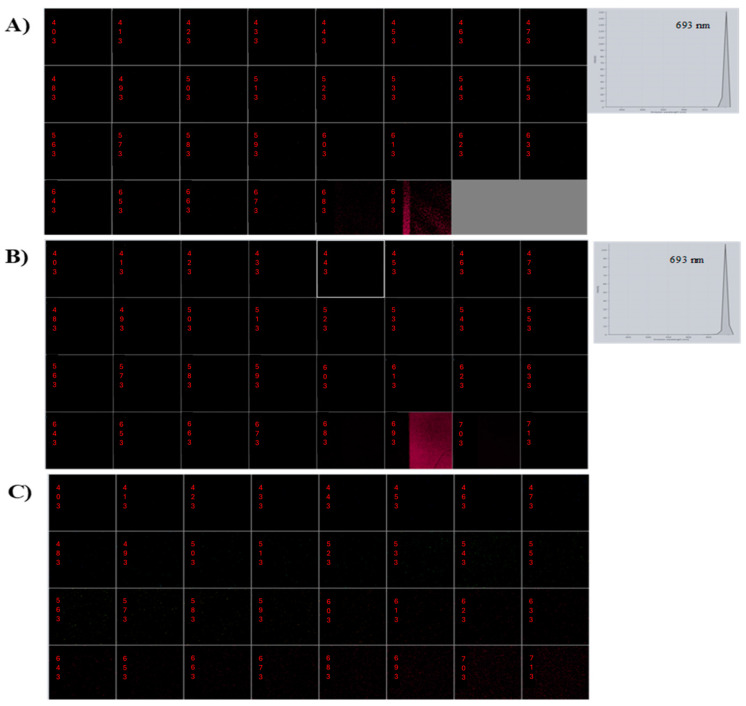
Lambda stack emission spectra: (**A**) affinin; (**B**) affinin + Nile Red; (**C**) *N*-isobutyl-decanamide.

To confirm whether alkamides co-localize with lipid droplets in the cotyledons of *H. longipes*, *MPFM* was employed. The emission lambda stack for pure affinin, collected at 10 nm intervals from 403 to 693 nm using a 780 nm excitation wavelength, is shown in Figure 4A. The linear unmixing technique enabled the generation of distinct emission or excitation fingerprints for affinin, revealing that this alkamide exhibits fluorescence emissions at 693 nm, corresponding to the red spectral region. This fluorescence, termed autofluorescence, is an intrinsic property displayed by certain compounds.

The analysis of the mix of NR–sunflower oil under MPFM revealed fluorescence emissions around 540 nm. Further, the emission lambda stack, collected at 10 nm intervals from 403 to 693 nm using a 780 nm excitation wavelength, exhibited fluorescence emissions at 693 nm, identical to when pure affinin was used (Figure 4B). NR is a highly effective stain for detecting intracellular lipid droplets via fluorescence microscopy, as it is highly soluble in triglyceride lipids and does not interact with other cellular structures [17], and in this case does not interfere with the emissions of the compound of interest.

To further confirm the specificity of affinin’s fluorescence emissions at 693 nm, the emissions of *N*-isobutyl-decanamide, a structural analog of affinin lacking the 2*E*,6*Z*,8*E* double bonds, were examined under identical conditions. No emissions were detected (Figure 4C), providing evidence that the fluorescence at 693 nm is specific to the 2*E*,6*Z*,8*E* double bonds of affinin and related alkamides. This suggests that the conjugated double bond in affinin’s acyl chain plays a crucial role in its intrinsic autofluorescence. Fluorescence signals measured in the blue (440 nm), green (520 nm), red (690 nm), and far-red (740 nm) regions are characteristic of plant metabolites. In plants, red and far-red fluorescence is typically associated with chlorophyll, while blue and green fluorescence is emitted by cinnamic acids and phenolic compounds covalently bound to cell walls [18]. Specifically, certain flavonoids and coumarins (chromones), pterins (pyridoxal coenzyme), some phenols (hydroxycinnamoyl acids), and alkaloids such as caffeine emit in the blue region (450–500 nm), while flavins and terpenoids emit in the green region (500–530 nm). Additionally, polyacetylenes, isoquinoline alkaloids, and other compounds emit in the yellow and orange regions, whereas anthocyanins and anthocyanidins emit in the red region [19,20,21].

**Figure 5:**
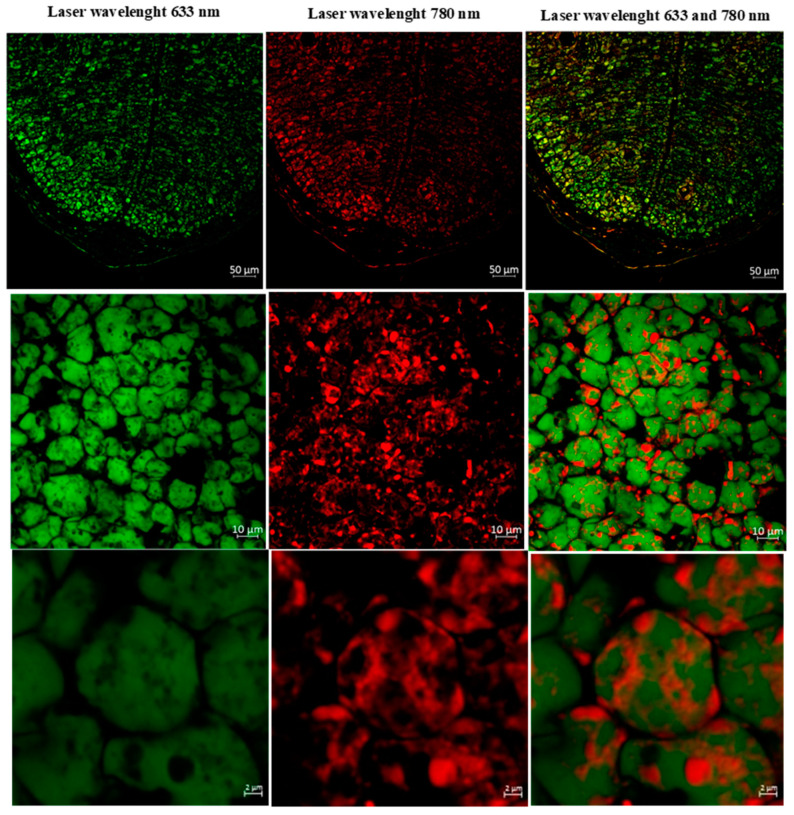
Autofluorescence of selected compounds present in a transversal slice of *H. longipes* cotyledons observed under multiphoton microscope. On the left, triglycerides stained with Nile Red. In the middle, the autofluorescence of alkamides (affinin). On the right, the overlapping of the two previous results.

After resolving the emission spectra of triglycerides, affinin, and Nile Red (NR), and confirming that the fluorescence observed at 693 nm is specific to affinin, NR-stained cotyledons were examined using *MPFM* to determine the localization of affinin. The emission lambda stack for affinin in cotyledons stained with NR, collected at 10 nm intervals from 403 to 693 nm using a 780 nm excitation wavelength, is presented in Figure 5. Multiphoton analysis revealed that affinin is localized in specific regions within the lipid bodies of *H. longipes* cotyledons (Figure 5. Given the non-polar nature of alkamides, it is reasonable to conclude that they are compartmentalized within lipid structures [15]. This experiment, combined with the data from GC-EIMS, confirms that alkamides such as affinin specifically accumulate within the lipid bodies of cotyledons.

Alkamides, particularly affinin, are known for their fungicidal, bactericidal, antiviral, and other antimicrobial activities [5,6,22]. This suggests a potential role for their accumulation in *H. longipes* seeds as a defense mechanism against microbial pathogens. Additionally, affinin and other alkamides have been reported to activate signaling pathways that confer resistance to certain plant pathogens [23]. Alkamides also act as signaling molecules that regulate meristematic activity and differentiation processes in non-producer plants [24]. Thus, their presence in *H. longipes* seeds may be associated with differentiation processes that occur during germination. However, the functional roles of alkamides within producer plants remain unclear. While their involvement in defense or germination is plausible, such interpretations are speculative. Further research is needed to elucidate the physiological roles of alkamides in the seeds and roots of *H. longipes*.

## 3. Materials and Methods

### 3.1. Plant Material

Flower heads of *H. longipes* containing mature cypselae were collected from the Puerto de Tablas municipality, Sierra Gorda, Xichú, Guanajuato, Mexico (21°14′20″ N, 100°05′19″ W, at an altitude of 2589 m above sea level). After collection, the material was brought to the laboratory, where cypselae were isolated and stored for subsequent analysis.

### 3.2. Reagents and Chemicals

Pure affinin was isolated from *H. longipes* root extract using column chromatography. *N*-isobutyl decanamide was prepared as previously described [10]. Nile Red (NR) was obtained from Sigma-Aldrich, Inc., St. Louis, MO, USA, (microscopy grade SKU72485), and all solvents used were HPLC grade, also purchased from Sigma-Aldrich, Inc. Food-grade sunflower oil was sourced from a local marketplace.

### 3.3. Cypselae Dissection and Ethanolic Extract Preparation

Approximately 50 mg of *H. longipes* cypselae was washed with distilled water, and the excess water was removed using absorbent paper. The cypselae were then immediately dissected using a scalpel under a Leica EZ4D stereoscope. Freshly dissected tissue from each cypsela fraction was macerated in absolute ethanol at a ratio of 1:10 (plant tissue/ethanol) at 4 °C for 24 h in the dark. The samples were then centrifuged for 5 min at 10,000 rpm, and the supernatant was collected. Each ethanolic extract was evaporated to dryness under a nitrogen stream at 25 °C and reconstituted with 50 µL of absolute ethanol. The extracts were stored at 4 °C until further analysis.

### 3.4. GC-EIMS Analysis of Alkamides

The ethanolic extract from each cypsela fraction was analyzed using GC-EIMS to identify alkamides. The analysis was conducted using an Agilent Technologies 7890A Gas Chromatograph (Santa Clara, CA, USA) coupled with an Agilent Technologies 5975e Electron Impact Ionization Mass Spectrometer. The system was operated in pulsed splitless injection mode with an injector temperature of 250 °C. Separation was achieved on an Agilent J&W DB-1MS UI capillary column (60 m × 320 µm × 1 µm), with helium as the carrier gas, at a constant flow rate of 1 mL/min [22]. Total ion chromatogram data were collected and analyzed using Mass-Hunter Qualitative Analysis software, version B.06.00 (Agilent Technologies, Inc.). Alkamides were identified by comparing their retention indices and mass spectra with those in the NIST MS Search 2.0 library (National Institute of Standards and Technology) [25]. The quantification of components was based on a standard curve generated with pure affinin.

### 3.5. Cypselae Transversal Slices Obtention

For microscopic analysis, cypselae were dissected using a scalpel under a Leica (Wetzlar, Germany) EZ4D stereoscope to obtain the complete structure of the cotyledons. Cross-sections (30 μm thick) were prepared from cypselae using a microtome.

### 3.6. Nile Red Stain for Determination of Lipid Droplets

Stock solutions of Nile Red (NR) (10 µg/mL) in acetone were prepared, stored, and protected from light. The dye was then directly applied to the tissue preparation as required, which was incubated for 10 min. Excess dye was removed by briefly rinsing the preparation in deionized water [17].

### 3.7. Lipid Droplets Staing and Observation

The cross-sections (30 μm thick) of cypselae were stained with Nile Red and observed under the 10× objective of an optical microscope B3-Professional Series DMB3-223 model (Thomas Scientific, Co., Swedesboro, NJ, USA); the microscopies were captured using the software Motic Images Plus 2.0 (Motic Inc., Hong Kong, China).

### 3.8. Localization of Affinin by Multiphoton Microscope Analysis

Cross-sections of cypselae (30 μm thick) were prepared for MPFM analysis. To obtain the autofluorescence fingerprint of affinin, the excitation and emission wavelengths were optimized. The specificity of affinin fluorescence was evaluated by comparing its signal with that of *N*-isobutyl-decanamide, a structural analog of affinin that lacks the 2*E*,6*Z*,8*E* double bonds, under identical optimized conditions. To further investigate the localization of alkamides within the cypselae structure, triglyceride-rich sunflower oil was mixed with an NR solution in a 5:1 ratio, and an emission lambda stack was collected at 10 nm intervals from 403 to 693 nm using a 633 nm excitation wavelength. Additionally, a mixture of pure affinin and NR solution was analyzed at the same 5:1 ratio. For these experiments, a multiphoton microscope system (LSM 880-NLO, Zeiss, Oberkochen, Germany), equipped with an infrared Ti Chameleon Vision II laser (COHERENT, Glasgow, UK) and an EC Plan-Neofluar 40×/1.30 Oil DIC M27 objective, was used. Optimal excitation for channel 1 (fluorescence) was achieved at λ = 633 nm, with detection in the 594–672 nm range using a photomultiplier tube (PMT) detector (Zeiss) for the triglyceride–NR mixture. For channel 2 (multiphoton fluorescence), optimal excitation was achieved at λ = 780 nm, with detection in the 584–684 nm range using a PMT detector, for affinin and the affinin–NR mixture.

### 3.9. Statistical Analysis

Alkamide quantification was performed in triplicate and the results are presented as the average of the individual replicates.

## 4. Conclusions

This study reports, for the first time, the presence of alkamides in the cypselae of *H. longipes*. GC-EIMS analysis revealed that the cypselae contain affinin and *N*-2-methylbutyl-2*E*,6*Z*,8*E*-decatrienamide, specifically localized in the cotyledons. Multiphoton microscopy further demonstrated that these alkamides are compartmentalized within specific structures in the cotyledons, known as lipid bodies. These lipid bodies serve as reservoirs of energy-rich triglycerides and play a role in seed germination. Further research is required to elucidate the ecological and physiological functions of affinin and related alkamides in the development of *H. longipes* seeds.

## Data Availability

Dataset available on request from the authors.
